# The short- and long-term effects of cognitive behavioral therapy on the glycemic control of diabetic patients: a systematic review and meta-analysis

**DOI:** 10.1186/s13030-023-00274-5

**Published:** 2023-05-08

**Authors:** Na Dong, Xiaowei Wang, Liu Yang

**Affiliations:** 1 The Affiliated Nanhua Hospital, Department of Endocrinology, Hengyang Medical School, University of South China, Hengyang, Hunan 421002 China; 2 Department of Endocrinology, People’s Hospital of Xinchang County, Zhejiang Province, Xinchang, 312500 China; 3 Department of Internal Medicine, Wuhan University Hospital, Wuhan, 430072 Hubei China

**Keywords:** Diabetes, CBT, Short-term, Long-term, Glycemic control, Meta-analysis

## Abstract

**Background:**

Glycemic control is an important issue in the treatment of diabetic patients. However, traditional methods, such as medication (the usual treatment), have limitations. Cognitive behavioral therapy (CBT) might be a useful option to help control the glycemic condition. The effects can be revealed by systemic review or meta-analysis of randomized clinical trials (RCT).

**Methods:**

A systematic search and a meta-analysis for the RCT were done of the short- and long-term effects of CBT on the glycemic control of diabetic patients in a comparison with the usual treatment. Nineteen RCT studies and 3,885 diabetic patients were enrolled in this meta-analysis. Subgroup analyses of types 1 and 2 diabetes and individual and group CBT were also performed.

**Results:**

Patients treated with CBT showed no significant difference in HbA1c when compared to the usual treatment within six months. However, CBT was more effective in reducing HbA1c when compared to usual treatment with at least six months of treatment duration [standardized mean difference: -0.44 (95% confidence interval (CI): -0.63 ~ -0.25), Z = 4.49]. Subgroup analysis of type 1 and 2 diabetic patients supported a long-term effect of CBT on glycemic control [standardized mean difference: -0.85 (95% CI: -1.19 ~ -0.10), Z = 2.23, standardized mean difference: -0.33 (95% CI:-0.47 ~ -0.19), Z = 4.52, respectively].

**Conclusions:**

CBT would be a useful option for improving the glycemic control of diabetic patients undergoing long-term treatment. The advantages of the long-term effects of CBT should be considered by clinicians and staff.

## Introductions

Diabetes mellitus (DM) is an important, chronic physical illness commonly seen in clinical practice. It is associated with physical and mental health problems, which contribute to comorbidity and increase the difficulty of treatment [1]. Glycated hemoglobin (HbA1c) is an important index for glycemic control. The maintenance of an ideal HbA1c is helpful in decreasing the complications of DM, such as peripheral neuropathy or nephropathy. In recent years, even with the improvement of diabetes medications, the glycemic control rate has not reached an ideal standard, which contributes to the microvascular and macrovascular complications of DM [2]. Because a third of type 2 DM patients do not achieve their target HbA1c, [3] methods to improve glycemic control are needed in addition to the medication treatment. Up to 50% of people with DM have poor mental health, which should not be neglected as a crucial comorbidity [4]. According to the American Diabetes Association, the standard care for people with DM should not focus only on the standard medication treatment. Self-management and psychological interventions might also play a useful role in improving glycemic control [1]. Self-regulation and self-integration are important for people with DM to achieve better psychological adjustment to their disease by fostering self-growth and resilience [5] Even with the differences in the underlying psychological mechanisms of type 1 and 2 DM, the importance of establishing better self-regulation and self-integration is crucial for both subtypes. A randomized trial of people with type 2 DM supported nurse-led motivational interventions that improve self-management, self–efficacy, quality of life, and glycemic control, which indicates that psychological intervention improves glycemic control through a bidirectional interaction between mind and body [6]. Diabetes-related stress contributes to the psychological burden and emotional responses of people with DM, influencing them via psychological domains, such as cognition and behavior [7].

From the above literature, the dysfunctional cognitive and behavioral responses of people with DM are crucial issues for improving their standard care. Cognitive-behavioral therapy (CBT) is commonly used in the treatment of dysfunctional cognitive beliefs and behaviors. It improves the management of DM through the replacement of dysfunctional cognition with a self-helping and realistic cognition [8]. The results of a recent network meta-analysis of adult patients with type 1 DM showed that CBT has potential treatment effectiveness, even without significant treatment effects of psychological interventions [9]. For type 2 DM, the latest network meta-analysis showed CBT to have a high treatment effect, even though there has been an impression of minimal clinical benefits for psychological interventions [3]. However, other meta-analyses showed that CBT might be beneficial for reducing the HbA1c and fasting blood sugar of people with DM, [10, 11] especially for people with DM comorbid with depression [12]. Another meta-analysis showed that CBT-based interventions improve glycemic control and depression symptoms with medium to large effects in people with type 1 and 2 DM. [13]. A previous meta-analysis reported that CBT might have short to medium-term treatment effectiveness for reducing HbA1c. The lack of long-term treatment effectiveness might be a disadvantage of CBT for the treatment of people with DM [14]. However, there may be overlap in the primary studies, and the heterogeneity of results could be due to differences in the methods used in these systematic reviews. For example, several meta-analytic studies enrolled studies of DM patients with comorbid depression [11, 12]. One meta-analysis focused only on patients with type 1 DM [9]. Several meta-analytic studies surveyed both type 1 and 2 DM [10, 13, 14]. One mixed pure DM patients and DM patients with comorbid depression [13]. CBT content, duration and frequency, concurrent medication treatment, and other variables were difficult to standardize in the previous meta-analytic studies. Therefore, it will be important to conduct meta-analyses with less bias than the enrolled studies and a more standardized group of DM patients.

From the above literature, we found heterogeneity of the meta-analysis results based on previous meta-analytic studies of people with DM. This study was done to clarify and confirm the treatment duration-related treatment effects of CBT on the glycemic control of people with DM. In this systematic review and meta-analysis we enroll only the randomized clinical trials of pure CBT for people with DM. In addition, we clarify the short- and long-term treatment effects of CBT for people with DM. Finally, we perform separate subgroup analyses of type 1 and 2 DM to confirm the short- and long-term treatment effects on glycemic control.

## Methods

### Inclusion criteria

To find all articles related to the effects of CBT for patients with DM, we searched and collected prospective RCT articles from the following databases: PubMed, ScienceDirect, EmBase, Web of Science, Scopus databases, Cumulative Index for Nursing and Allied Health Literature (CINAHL), and the Cochrane Central Register of Controlled Trials (CENTRAL). The keywords included “cognitive behavioral therapy”, “cognitive”, “behavioral”, “diabetes”, “diabetic”, “randomized”, “clinical”, “controlled”, “trial”, “HbA1c”, “glycemic control” or “outcome”, “comparison”, “versus”, “treatment”, “usual treatment”. The article search was limited to those published or e-published online before July 2022. The date of our last search of PubMed, ScienceDirect, EmBase, and Web of Science was June 30, 2022. The date of our last search of Scopus databases, CINAHL, and CENTRAL was June 29, 2022.

The inclusion criteria were (1) Comparisons between CBT and usual treatment for diabetic patients. (2) Studies with an outcome of glycemic control and specific for HbA1c. (3) Studies with detailed data on the outcome of glycemic control and with data specific for HbA1c. (4) Clinical trials with a randomized design. (5) Articles written in English that were published in the journals of the science citation index database. All authors (DN, WX, and YL) participated the study and DN and WX performed the article selection process according to the inclusion criteria.

### Risk of bias assessment

The Cochrane Handbook for Systematic Reviews and Interventions gave us the direction to conduct the current meta-analysis [15]. The risk of bias was evaluated by the following dimensions: bias arising from the randomization process, bias due to deviations from intended interventions, bias due to missing outcome data, bias in measurement of the outcome, and bias in selection of the reported result [16]. The results are reported according to the preferred reporting items for systematic reviews and meta-analyses (PRISMA) guidelines [17].

### Extraction and critical appraisal of data

The data from the enrolled articles was collected by DN and WX. First, the baseline HbA1c of diabetic patients in the CBT and usual treatment groups. Second, the short-term (less than 6 months) endpoint HbA1c after CBT or usual treatment. Third, the long-term (more than 6 months) endpoint HbA1c after CBT or usual treatment. The rationale for our classification of short- and long-term was that we aimed to find the treatment duration-related effects of CBT on the HbA1c of people with DM. Fourth, the short- and long-term endpoint HbA1c of the CBT and usual treatment groups of patients with different subtypes of diabetes (types 1 and 2 diabetes). DN and WX assessed the abstracts to screen the articles and independently evaluated the full text version of the selected citations. The clinical outcome data was independently collected from text, tables, and figures of the enrolled articles by the two reviewers. The enrolled articles basically had data on the short-term endpoint HbA1c, long-term endpoint HbA1c, or subtypes of diabetes in the full text content. All authors (DN, WX, and YL) participated a collaborative review to resolve any discrepancies and to achieve agreement (kappa = 0.8) and participated in the review of the final results. The GRADE (grading of recommendations, assessment, development, and evaluation) approach was used by DX and YL to assess the certainty of evidence.

### Meta-a and statistical analysis

The current meta-analysis was analyzed using the Cochrane Collaboration Review Manager Software Package (Rev Man Version 5.4). The CBT and usual treatment groups were compared to find which treatment would be superior for the glycemic control of diabetic patients. The overall effect size of the short- and long-term HbA1c endpoints were calculated as the weighted average of the inverse variance for the study-specific estimates. The weighted standardized mean difference was used to estimate numerical variables for the continuous variables. Heterogeneity was estimated using the χ^2^ distribution test, Higgins I^2^ index, Cochran's Q, and Tau square test. The synthesized results were conducted by pooling the data and using a random effects model meta-analysis. Subgroup analyses of the subtypes of diabetes were performed to confirm the treatment effects of CBT or usual treatment on glycemic control. In addition, forest plotting was done to demonstrate if the meta-analysis would favor CBT or usual treatment for improving the glycemic control of diabetes. Finally, a test for overall effect was done to calculate the Z and p-values for significance. The above meta-analysis steps were performed by DN and YL.

## Results

### Characteristics of the studies enrolled

**Fig. 1:**
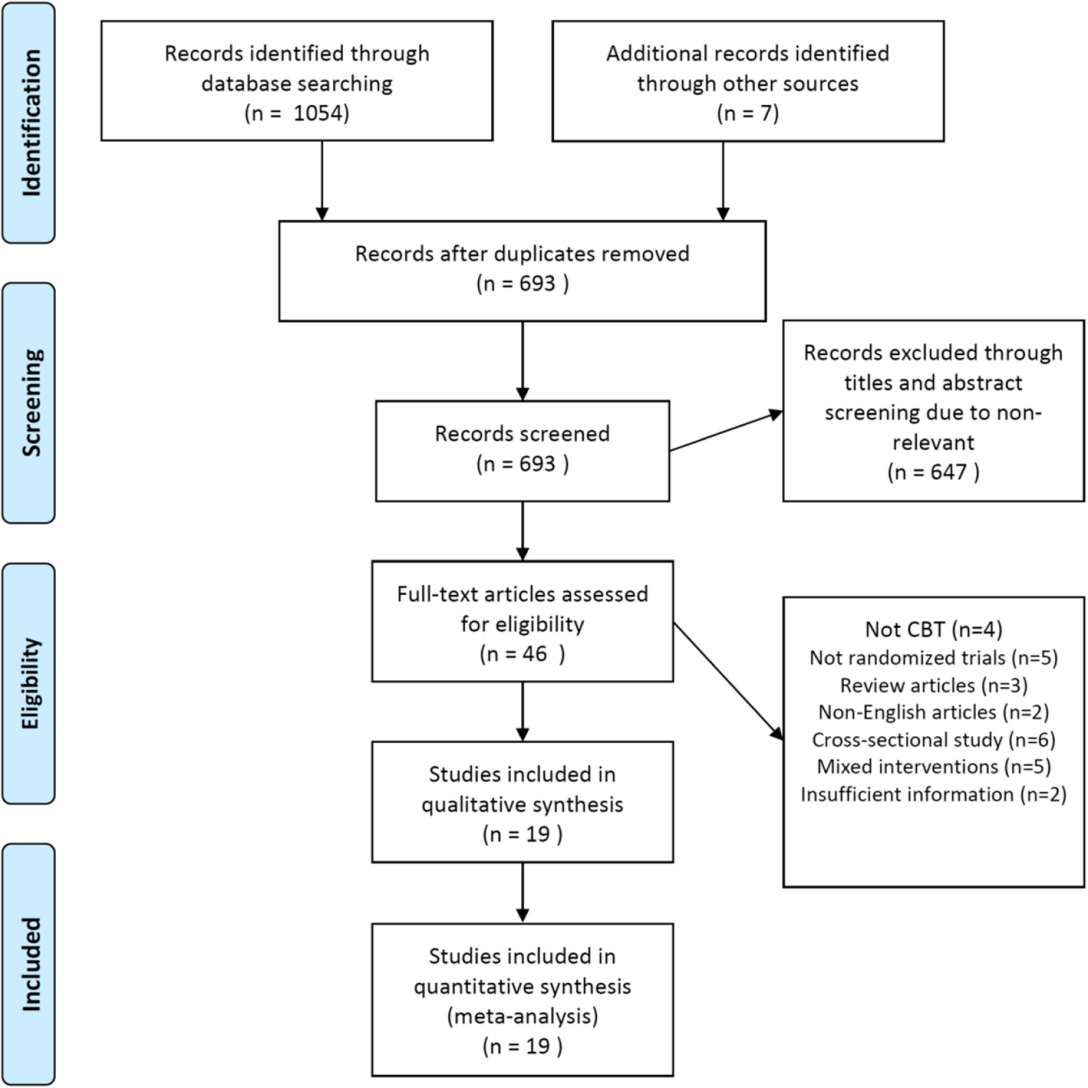
Search and selection flowchart. The current meta-analysis followed the PRISMA guidelines and used abstract and title selection to identify the potentially relevant literature and then to screen it. The full text of the screened literature was assessed to find studies eligible for inclusion in the final meta-analysis

**Fig. 2:**
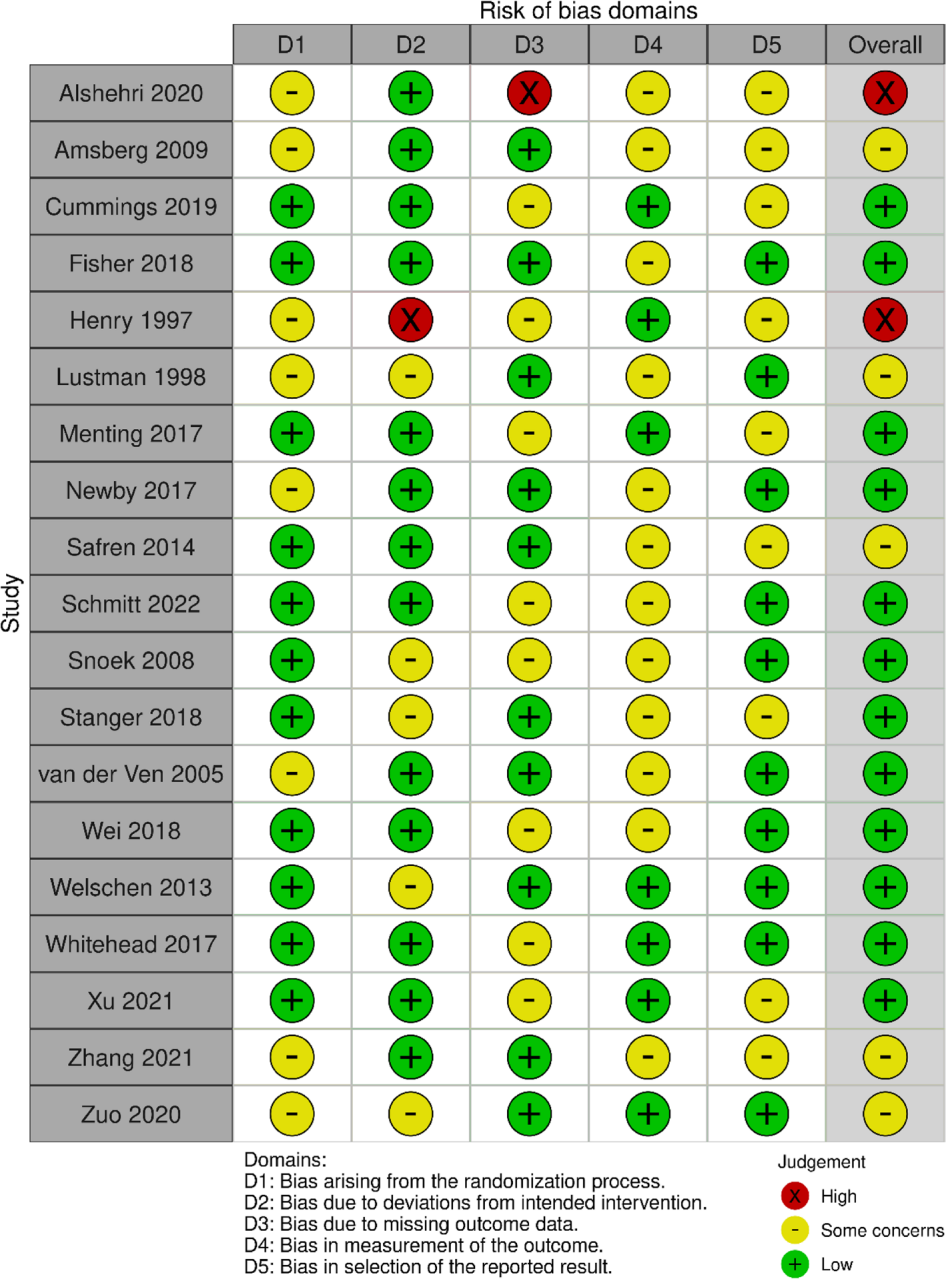
Risk of bias assessment for short-term studies. The risk of bias assessment for short-term studies was performed on the enrolled articles according to the risk of bias assessment updated version (ROB v2)

**Fig. 3:**
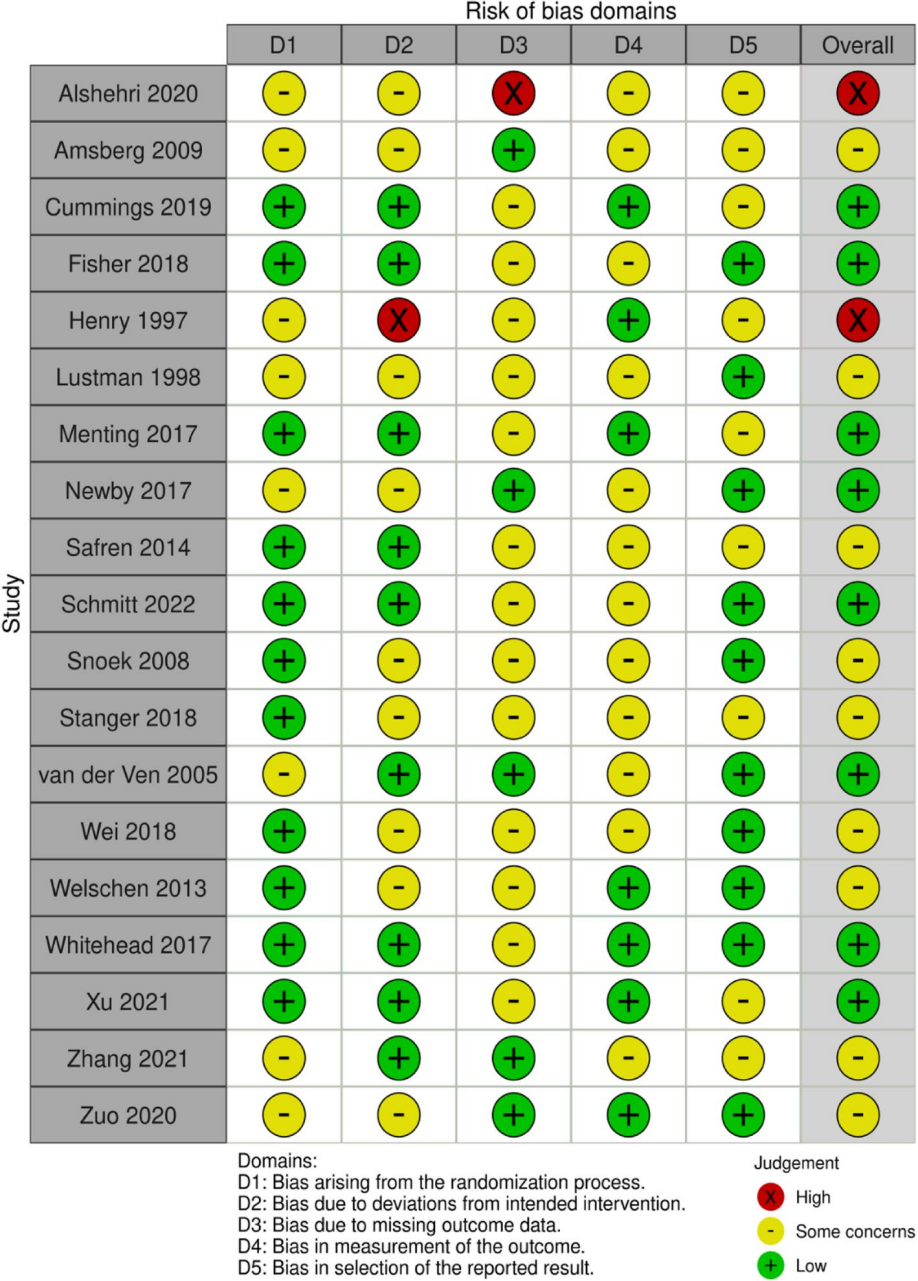
Risk of bias assessment for long-term studies. The risk of bias assessment for long-term studies was performed on the enrolled articles according to the risk of bias assessment updated version (ROB v2)

The article selection process is presented as a PRISMA flow diagram in Fig. 1. The eligibility of the 46 screened articles was assessed according to the content of the full text after the article selection process: 27 were eliminated (Fig. 1), leaving the data of 19 available for the qualitative analysis [18–36]. The characteristics of the enrolled 19 studies are summarized in Table 1. In addition, the risk of bias assessment for the short-term studies is presented in Fig. 2 and the risk of bias assessment for the long-term studies is presented in Fig. 3.

**Table 1:** Summary of enrolled studies

	Subjects (CBT vs usual treatment)	CBT content vs usual treatment	Blinded	Outcome of interest
Alshehri 2020 (USA) [32]	Type 2 DM 14 (40–75 years old) vs 14 (40–75 years old)	CBT for insomnia vs Health education Duration: 50 days	Unblinded	glycemic control,diabetes self-care behavior, and fatigue
Amsberg 2009 (Sweden) [22]	Type 1 DM 40 (22 M, 18 F, 41.1 ± 11.7 years old) vs 39 (16 M, 23 F, 41.4 ± 12.9 years old)	CBT-based Intervention Vs Usual treatment (including Continuous monitoring of blood glucose level) Duration: 48 weeks	Unblinded	HbA1c, self-care behaviors and psychosocial factors
Cummings 2019 (USA) [31]	Type 2 DM 67 (14 M, 53 F, 51 ± 9 years old) vs 72 (17 M, 55 F, 53 ± 9 years old)	A tailored CBT intervention plus lifestyle counseling and standard medical care vs usual treatment Duration: 12 months	Unblinded	HbA1c, regimen-related distress, depression, self-care behaviors, medication adherence, systolic blood pressure, body weight
Fisher 2018 (USA) [28]	Type 1 DM 149 (44 M, 105 F, 47.3 ± 14.5 years old) vs 152 (49 M, 103 F, 42.8 ± 15.1 years old)	OnTrack used a variety of scenarios and exercises, based on emotion regulation, that focused on ways to deal with the emotional side of diabetes and how to develop personalized emotion management techniques vs Know It included a diabetes update of key factors regarding the causes and management of T1D, based on a UCSF Diabetes Education Program. Duration: 3 months and 9 months	Unblinded	Diabetes distress, powerlessness, diabetes management, hypoglycemic distress, negative social perceptions, eating distress, physical distress, friend and family distress, HbA1c
Henry 1997 (Australia) [18]	type 2 DM 10 (4 M, 6 F) vs 9 (5 M, 4 F) 59.8 years old (47–74 years old)	CBT (6 weekly 1.5-h sessions conducted in 2 small groups) vs waiting list Duration: 6 weeks	Unblinded	HbA1c, Blood glucose, State anxiety, Hassless frequency, perceived stress, coping ability, Beck Depression Inventory
Lustman 1998 (USA) [19]	Type 2 DM with major depression 20 (8 M, 12 F, 53.1 ± 10.5 years old) vs 22 (9 M, 13 F, 56.4 ± 9.7 years old)	Individual CBT (1 h weekly for 10 weeks) vs diabetes education Duration: 10 weeks	Unblinded	Depression severity, Depression remission, HbA1c
Menting 2017 (Netherlands) [25]	Type 1 DM 60 (23 M, 37 F, 44.4 ± 12.1 years old) vs 60 (23 M, 37 F, 42.9 ± 12.5 years old)	The CBT intervention (Dia-Fit) was given for 5 months in blended form, consisting of face-to-face and web-based sessions vs waiting list Duration: 5 months	Unblinded	Fatigue severity, Functional impairment, HbA1c, glucose variability
Newby 2017 (Australia) [26]	Type 1&2 DM 41 (8 M, 33 F, 43.5 ± 13.3 years old) vs 49 (18 M, 31 F, 49.3 ± 11.5 years old)	Internet-based CBT (participants completed 6 automated cartoon-style web-based lessons teaching CBT skills over 10 weeks, with a minimum wait-time of 5 days between lesson) vs usual treatment Duration: 3 months	Unblinded	HbA1c, problem areas in diabetes scale, Kessler 10-item psychological distress scale, short form 12-item mental health subscale, short form 12-item physical health subscale, generalized anxiety disorder 7-item scale, patient health questionnaire 15-item somatization scale
Safren 2014 (USA) [24]	Type 2 DM 45 (22 M, 23 F, 55.44 ± 8.72 years old) vs 42 (22 M, 20 F, 58.31 ± 7.41 years old)	CBT vs usual treatment Duration: 4 months	Single blinded	HbA1c the effectiveness of CBT for medicine adherence and depression in patients
Schmitt 2022 (Germany) [36]	Type 1&2 DM 131 (56 M, 75 F, 45.2 ± 13.9 years old) vs 129 (62 M, 67 F, 44.9 ± 15.1 years old)	Stepped CBT (step 1: Five sessions (90 min each) of diabetes-specific CBT group step 2: 6 to 12 sessions of single CBT for depression (50 min each) via telephone step 3: Referral for outpatient depression treatment by psychotherapist and/or psychiatrist vs usual treatment Duration: 6 months and 12 months	Unblinded	HbA1c, rate of meaningful depression reduction, problem areas in diabetes scale, Kessler 10-item psychological distress scale, short form 12-item mental health subscale, short form 12-item physical health subscale, generalized anxiety disorder 7-item scale, patient health questionnaire 15-item somatization scale, interleukin-6,18,1 receptor anatagonist
Snoek 2008 (Netherlands) [21]	Type 1 DM 45 (18 M, 27 F, 38.1 ± 9.7 years old) vs 41 (14 M, 27 F, 37.4 ± 11.1 years old)	group CBT vs usual treatment Duration: 12 months	Unblinded	HbA1c, Depression scales
Stanger 2018 (USA) [29]	Type 1 DM 30 (19 M, 11 F, 15.2 ± 1.4 years old) vs 31 (16 M, 15 F, 14.9 ± 1.5 years old)	Web delivered CBT (incentives, brief motivational interviewing/CBT and parent contingency contracting sessions, and working memory training all delivered over the internet) vs usual treatment Duration: 6 months and 12 months	Unblinded	HbA1c, self-monitoring of blood glucose Parent monitoring, visual spatial working memory scale, Diabetes Family Conflict Scale, Stroop color-word interference
van der Ven 2005 (Netherlands) [20]	Type 1 DM 52 vs 55	Group CBT vs usual treatment	Unblinded	HbA1c, confidence in diabetes self-care scale, problem areas in diabetes questionnaire, center for epidemiological studies depression scale
Wei 2018 (UK) [30]	Type 1 DM 43 (19 M, 24 F, mean 13.2 years old) vs 42 (19 M, 23 F, mean 14.1 years old)	CBT (six 1-to-1 weekly sessions with single follow-up sessions at 6 and 12 months) vs usual treatment Duration: 3, 9, 12, 24 months	Unblinded	HbA1c, diabetes family behaviour scale, diabetes’ quality of life for youths, locus of control,well-being questionnaire
Welschen 2013 (Netherlands) [23]	Type 2 DM 76 (45 M, 31 F, 60.5 ± 9.4 years old) vs 78 (50 M, 28 F, 61.2 ± 8.8 years old)	CBT vs Usual treatment Duration: 6 months	Unblinded	The effects of CBT for reducing the risk of CHD, HbA1c, total cholesterol blood pressure, depression scale
Whitehead 2017 (Australia) [27]	Type 2 DM 39 (17 M, 22 F, 56.1 ± 6.9 years old) vs 45 (50 M, 28 F, 61.2 ± 8.8 years old)	CBT-based acceptance and commitment therapy vs usual treatment Duration: 3 and 6 months	Double blinded	HbA1c, anxiety scale, depression scale diabetes treatment satisfaction questionnaire
Xu 2021 (China) [34]	Type 2 DM 597 (186 M, 411 F, 63.81 ± 9.94 years old) vs 611 (218 M, 393 F, 62.91 ± 9.59 years old)	Group CBT (10 sessions in 10 consecutive days. Each session lasted 40–50 min and was followed by a 10- to 15-min discussion) vs usual treatment Duration: 2, 6 and 12 months	Unblinded	HbA1c, generalized anxiety disorder 7-item scale, patient health questionnaire
Zhang 2021 (China) [35]	Type 2 DM 568 (198 M, 370 F, 61.58 ± 9.17 years old) vs 574 (191 M, 383 F, 61.83 ± 8.64 years old)	CBT vs Usual treatment Duration: 2, 6 and 12 months	Unblended	HbA1c, Pittsburgh sleep quality index
Zuo 2020 [33]	Type 2 DM 94 (32 M, 62 F, 63.9 ± 10.2 years old) vs 93 (29 M, 64 F, 61.7 ± 10.4 years old)	CBT (a 2-month involving seven sessions. Each session lasted 40–50 min, and was followed by a 10–15-min discussion) vs usual treatment Duration: 2 and 6 months	Double blinded	HbA1c, Pittsburgh sleep quality index, diabetes-specific quality of life scale

### Meta-analysis results for the effect of CBT vs usual treatment on HbA1c over the short-term

Of the 19 articles enrolled, 13 had outcome data for short-term HbA1c. The mean difference in the short-term HbA1c of the CBT and usual treatment groups in the random effects model was -0.05 [95% confidence interval (CI): -0.16 ~ 0.06], without significance (test for overall effect Z = 0.92, *p* = 0.36). Low heterogeneity was noted [Tau^2^ = 0.00, Chi^2^ = 8.90, df = 12 (*p* = 0.71), I^2^ = 0%].

### Meta-analysis results for the long-term HbA1c of CBT vs usual treatment

**Fig. 4:**
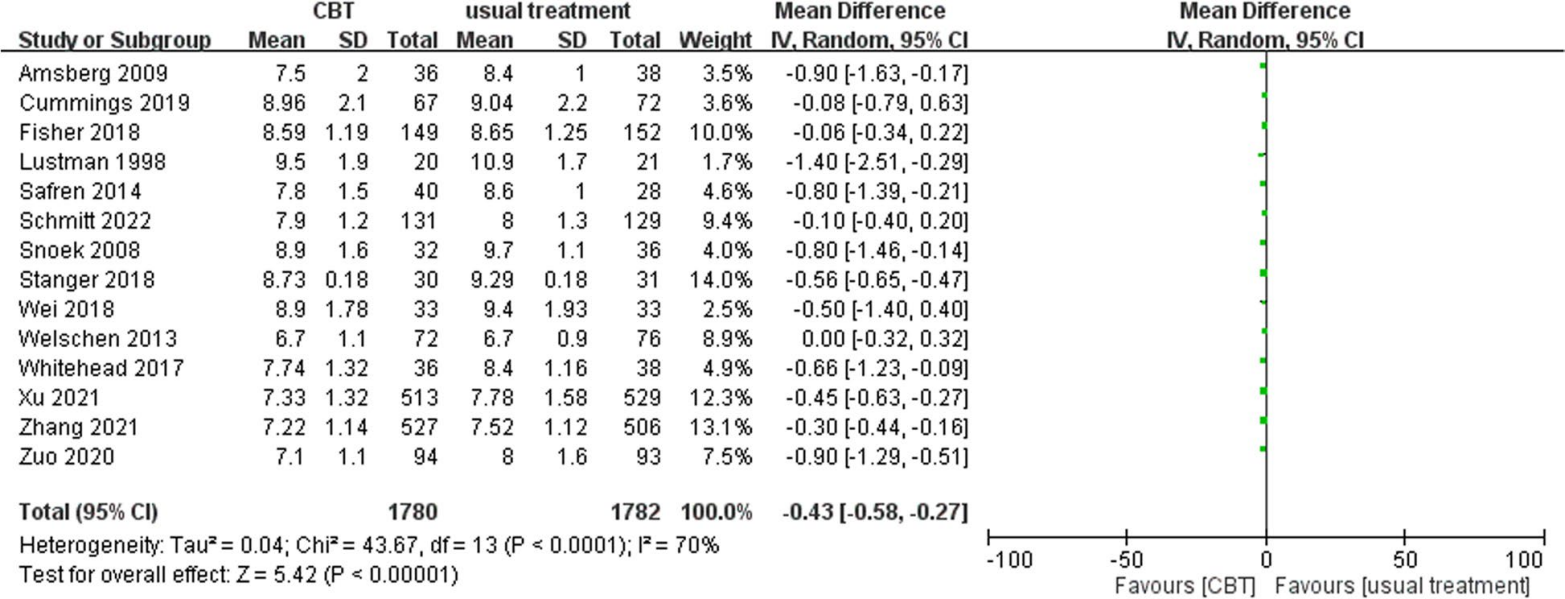
Forest plot of the meta-analysis results for long-term HbA1c [CBT vs usual treatment]. The CBT group had significantly lower long-term HbA1c than the usual treatment group. High heterogeneity was noted

Of the 19 articles, 13 had outcome data of the long-term endpoint HbA1c. The mean difference in the long-term HbA1c of the CBT group and usual treatment groups in the random effects model was -0.43 (95% CI: -0.58 ~ -0.27). Long-term HbA1c was significantly lower in the CBT group than in the usual treatment group (test for overall effect Z = 5.42, *p* < 0.00001). High heterogeneity was noted [Tau^2^ = 0.04, Chi^2^ = 43.67, df = 13 (*p* < 0.0001), I^2^ = 70%] (Fig. 4). The statistical heterogeneity might be a consequence of clinical heterogeneity (variability in the participants, interventions, and/or outcomes) or methodological heterogeneity (variability in study design, outcome measurement tools, and/or risk of bias) among the selected RCTs (https://training.cochrane.org/handbook/current/chapter-10#section-10-10). Due to the lack of patient-level data, we performed the following subgroup analyses of types 1 and 2 DM to survey for potential clinical heterogeneity.

### Analysis of the effect on HbA1c of long-term CBT vs usual treatment for the type 1 DM subgroup

**Fig. 5:**
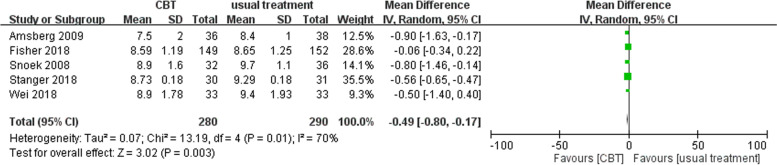
Analysis of long-term HbA1c in the subgroup of people with type 1 DM [CBT vs usual treatment]. The CBT group had significantly lower long-term HbA1c than the usual treatment group. High heterogeneity was noted

The mean long-term difference in the HbA1c of the CBT and usual treatment groups was -0.49 (95% CI: -0.80 ~ -0.17) for the type 1 DM subgroup, which indicates a significantly lower long-term endpoint HbA1c for the CBT group than was found for the usual treatment group (test for overall effect Z = 3.02, p = 0.003). High heterogeneity was noted [Tau^2^ = 0.07, Chi^2^ = 13.19, df = 4 (*p* = 0.01), I^2^ = 70%] (Fig. 5).

### Analysis of the effect on HbA1c of long-term CBT vs usual treatment for the type 2 DM subgroup

**Fig. 6:**
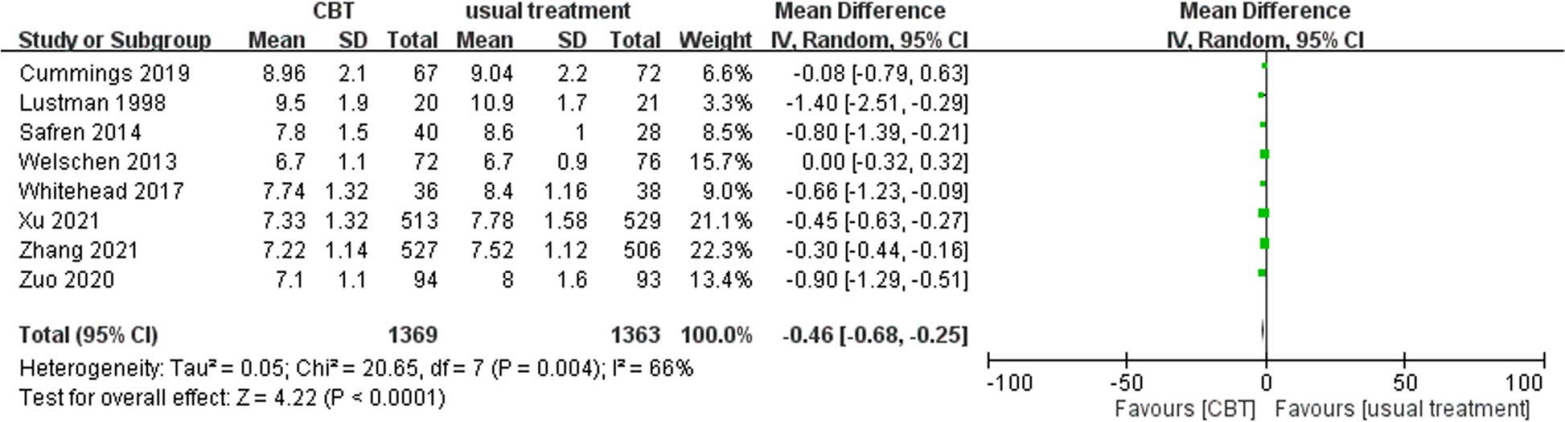
Analysis of long-term HbA1c in the subgroup of people with type 2 DM [CBT vs usual treatment]. The CBT group had significantly lower long-term HbA1c than the usual treatment group. Moderate heterogeneity was noted

The mean long-term difference in the HbA1c of the CBT and usual treatment groups was -0.46 (95% CI: -0.68 ~ -0.25) for the type 2 DM subgroup, which indicates significantly lower long-term HbA1c in the CBT group than was found in the usual treatment group (test for overall effect Z = 4.22, *p* < 0.0001). High heterogeneity was noted [Tau^2^ = 0.05, Chi^2^ = 20.65, df = 7 (*p* = 0.004), I^2^ = 66%] (Fig. 6).

### Assessments of certainty (or confidence) in the body of evidence

Our evaluation found moderate confidence in the effect estimate.

## Discussion

In the current meta-analysis, the treatment effect of CBT demonstrates a long-term effect on HbA1c. The significantly lower HbA1c after at least six months of study duration indicates that CBT expresses its potential and augmentation role in improving the glycemic control of diabetic patients. The subgroup analysis of people with type 1 and 2 DM over the long-term supports the potential and augmentation role of CBT for relieving the hyperglycemic status; however, the short-term effect was not significant for CBT on the glycemic control of diabetic patients. The subgroup analyses of types 1 and 2 DM individually also supported a lack of significant treatment effect over the short-term on endpoint HbA1c. Our meta-analysis results indicate that the augmentation and potential effects of CBT on the glycemic control of diabetic patients might be significant only over the long-term (more than six months).

The current meta-analysis failed to find a significant short-term effect of CBT on HbA1c, possibly for the following reasons. First, the definition of short-term effect in current meta-analyses is within six months, which might be different from the definition of previous meta-analyses. Second, the RCTs enrolled in the current meta-analysis might be more up-to-date than previous meta-analyses. However, future comprehensive review and meta-analysis will be needed to confirm the short-term effect of CBT on the HbA1c of people with DM.

Our results also corresponded to those of a previous meta-analysis that showed that CBT is effective in reducing fasting blood glucose, with a long-term effect [12]. Another meta-analysis of psychological interventions showed only that CBT has the potential to be effective for adult patients with type 1 DM [9]. In addition, a meta-analysis of people with type 2 DM showed that psychological interventions can effectively reduce HbA1c and that CBT has the highest probability of intervention effectiveness [3]. A recent meta-analysis of CBT-based intervention (not pure CBT) found improved glycemic control, with a moderate to large effect size, however, the heterogeneity of CBT may have limited the interpretations [13]. Another meta-analysis of diabetic patients with depression reported that CBT might be most effective for glycemic control within six months [11]. A meta-analysis focused on the content and style of CBT showed that, in group-based and face-to-face methods, psycho-education, behavioral, cognitive, goal-setting, and homework assignment strategies all significantly reduced HbA1c, which indicates that CBT of different content and styles have a similar effect on glycemic control [10]. These meta-analyses supported the importance of the augmentation therapy role of CBT for glycemic control and psychological symptoms [37]. The effect of CBT on glycemic control might be related to the reduction of negative thoughts, attitudes, and beliefs regarding diabetes and might lead to a change in dysfunctional self-care behaviors and improve subsequent glycemic control [14].

However, all these meta-analyses demonstrated only that the effectiveness of CBT on glycemic control might be limited to medium- or short-term duration [14]. Uchendu et al. stated that the lack of long-term effects might be due to not so many studies at that time having focused on the long-term treatment duration (2017). The long-term effects might appear if more long-term treatment studies of CBT are enrolled, [14] which might explain the reason for the long-term effects of CBT on people with DM in the current meta-analysis. These previous meta-analyses did not find a significant reduction of HbA1c over the long-term. Our meta-analysis enrolled more long-term studies (nine done since 2017) and showed a significant long-term effect on HbA1c, which corresponds to Uchendu et al.’s conclusion about the lack of significant long-term effects in previous meta-analyses. In the current meta-analysis, a significant short-term effect on glycemic control was not found: only a long-term effect was significant. Our subgroup analysis of people with type 1 and 2 DM also supports the significant role of long-term CBT in reducing HbA1c. Long-term CBT may be associated with gray matter changes of brain structures, such as the amygdala. It might reduce self-referential criticisms and might be helpful in reducing dysfunctional belief and behaviors [38]. The connectivity between the prefrontal cortex and amygdala might also be modulated by CBT, which might also reduce dysfunctional belief and behaviors [39]. Therefore, future study to clarify the short and long-term effects of CBT on glycemic control will be necessary.

There are several limitations to the current meta-analysis. First, even though we enrolled 19 studies with a relatively large sample size, only 13 with 3,296 total participants reported short-term effects and 14 with 3,562 participants reported long-term effects. The sample size is somewhat small, which might limit the generalization of our results. The enrollment of more randomized studies with more participants will be necessary in the future. Second, the variable content of CBT limits the results. This variation includes the frequency, intensity, duration, session content, total number of sessions, group or individual, real face-to-face or web-delivered, training of the therapists, and directions of CBT. These variables might interfere the solidness of evidence for CBT. Future studies with a more consistent structure and duration of CBT will be helpful in reducing such confounding factors. Third, the types of DM and the subject’s demographic data limit the interpretations of the current meta-analysis. Our meta-analysis enrolled studies of people with type 1 and 2 DM. Even the subgroup analysis of type 1 and 2 DM separately showed consistent, significant results for the long-term effects of CBT on glycemic control. However, the pathophysiology and epidemiology of type 1 and 2 DM are different, which might have contributed to different responses to CBT. In addition, many adolescents have type 1 DM, as was reported in several enrolled studies. The variability of the age of the diabetic patients in the current meta-analysis means that our results must be interpreted with caution. In addition, the predominance of female patients enrolled in the studies may have led to a gender-specific influence on the current results. Fourth, the lack of patient-level data may also limit the interpretation of our results due to the lack of a full evaluation of patient-level covariates in cross comparisons. It is impossible to confirm a possible subgroup effect related to patient age or to explore the impact of variation in the disease condition of the patients in the enrolled studies. Fifth, due to the lack of such data in most studies, the current meta-analysis compares only the difference of the HbA1c endpoints, not the change of HbA1c. Future studies of changes in HbA1c by CBT for diabetic patients are warranted. Sixth, our method of assignment to intervention may have led to bias in our results. For interventions over a long period, only subjects who did not drop out (good adherence patients) are included in the analysis. The intention-to-treat effect was not properly evaluated as a result, which might influence the interpretation. Seventh, the variations of the interventions and significant statistical heterogeneity might influence the validity of conducting a meta-analysis under such conditions. However, because we used the random effects model, it should be appropriate due to its ability to capture uncertainty resulting from heterogeneity among studies [40].

## Data Availability

Can be obtained from the corresponding author under a reasonable request.

## Abbreviations

CBT: Cognitive behavioral therapy
DM: Diabetes mellitus
HbA1c: Hemoglobin A1c
RCT: Randomized clinical trial
